# Discrimination of cryptic species: *Tabanus triangulum* and *Tabanus occidentalis* (Diptera: Tabanidae) differ in size and shape

**DOI:** 10.1590/S1984-29612024028

**Published:** 2024-06-17

**Authors:** Gratchela Dutra Rodrigues, Boaventura Lobo Centeno, Diuliani Fonseca Morales, Rafaela de Freitas Rodrigues Mengue Dimer, Caroline da Silva Cavalheiro, Tiago Kütter Krolow, Mauricio Osvaldo Moura, Rodrigo Ferreira Krüger

**Affiliations:** 1 Programa de Pós-graduação em Biodiversidade Animal – PPGBDiv, Universidade Federal de Pelotas – UFPel, Pelotas, RS, Brasil; 2 Programa de Pós-graduação em Entomologia – PPGEnt, Universidade Federal de Pelotas – UFPel, Pelotas, RS, Brasil; 3 Programa de Pós-graduação em Microbiologia e Parasitologia – PPGMPar, Universidade Federal de Pelotas – UFPel, Pelotas, RS, Brasil; 4 Laboratório de Ecologia de Parasitos e Vetores, Universidade Federal de Pelotas – UFPel, Pelotas, RS, Brasil; 5 Programa de Pós-graduação em Biodiversidade, Ecologia e Conservação – PPGBEC, Universidade Federal de Tocantins – UFT, Porto Nacional, TO, Brasil; 6 Departamento de Zoologia, Universidade Federal do Paraná – UFPR, Curitiba, PR, Brasil

**Keywords:** Horse flies, wing geometric morphometric, mechanical vector, Mutucas, morfometria geométrica de asas, vetor mecânico

## Abstract

Horse fly females (Diptera, Tabanidae) are hematophagous and can vector pathogens that affect livestock. Complexes of cryptic species are common in Tabanidae, as exemplified by some species of *Tabanus*, including *Tabanus triangulum* and *Tabanus occidentalis*, both prevalent in the Southern region of Brazil. In this study, geometric morphometrics were employed to ascertain the wing venation in species identification. It was demonstrated that this tool effectively differentiates *T. triangulum* from *T. occidentalis* in the coastal plain of Rio Grande do Sul state, situated within the Pampa biome. The results indicate that *T. triangulum* and *T. occidentalis* occupy distinct regions of the morphological space, allowing their precise identification through geometric morphometrics, which is fast, affordable, and easy to implement.

## Resumo

Fêmeas de mutucas (Diptera, Tabanidae) são hematófagas e podem ser vetores de patógenos que afetam animais de criação. Complexos de espécies crípticas são comuns em Tabanidae, como exemplificado por algumas espécies de *Tabanus*, incluindo *Tabanus triangulum* e *Tabanus occidentalis*, ambas prevalentes na região Sul do Brasil. Neste estudo foi utilizada morfometria geométrica para avaliar a venação das asas na identificação de espécies. Foi demonstrada que essa ferramenta diferencia, efetivamente, *T. triangulum* de *T. occidentalis* na Planície Costeira do Rio Grande do Sul, situada no bioma Pampa. Os resultados indicam que *T. triangulum* e *T. occidentalis* ocupam regiões distintas do espaço morfológico, permitindo sua identificação precisa por meio da morfometria geométrica, a qual é rápida, acessível e fácil de implementar.

## Introduction

Tabanidae (horse flies) comprise approximately 4,525 species (Pape & Thompson, 2023), of which 27% are found in the Neotropical region (Henriques et al., 2012). Several species are mechanical vectors of pathogens to humans and animals (Baldacchino et al., 2014; Taioe et al., 2017; Bilheiro et al., 2019; Rodrigues et al., 2021; 2022; Ramos et al., 2023). Tabanid studies have focused on females because, since they need a blood meal before ovipositing, they are the vectors of pathogens (Barros, 2001; Rafael & Charlwood, 1980).

In the Pampa biome, covering Argentina, Uruguay, and southern Brazil, there are records of 46 horse fly species from 16 genera (Coscarón & Papavero, 2009; Krüger & Krolow, 2015; Lucas et al., 2020). *Tabanus*, boasting 1,367 described species (Pape & Thompson, 2023), is the most diverse genus of the family in the world. Among the Pampa horse flies, two species, *Tabanus occidentalis* Linnaeus, 1758 and *Tabanus triangulum* Wiedemann, 1828, are often difficult to separate due to their morphological similarities.

The species *T. occidentalis* is widely distributed in the Neotropics and is common in various vegetation types from Mexico to Argentina (Coscarón & Papavero, 2009). This polymorphic species shows variations in color, size, and the shape of the frons, callus, and antennae. In a review of the group, Fairchild (1983) considered some morphological variants as valid species or varieties (Fairchild & Burger, 1994), but later the individual species were synonymized with *T. occidentalis* by Coscarón & Papavero (2009). In contrast, *T*. *triangulum* has a more restricted distribution, Brazil, Paraguay, Uruguay, Bolivia, and Argentina (Coscarón & Papavero 2009; Guimaraes et al., 2016). Although the species shares habitats with *T. occidentalis* in the Atlantic Forest and Pampa (Krüger & Krolow, 2015; Guimaraes et al., 2016), it is more prevalent in Pampa areas. Although no varieties have been proposed for *T. triangulum*, variations in color, shape, and structure size have been observed in this species.

Besides the polymorphism and overlap in the distribution of both species, they also share many similarities, leading to frequent misidentifications (Silva, 2016) by researchers who are not specialized in the group. These similarities include body size and general coloring, including the color pattern of the tibia (Figures 112, 1.5-1.6), the shape of the frontal callus (Figures 113, 1.7), the frontal indices (FI) and divergence (ID) (Figures 113, 1.7), and the shape of the antenna and palp (Figures 114, 1.8). Despite the overlaps, some characters, when examined by trained specialists, allow for the differentiation of taxa. The features assisting in differentiation include the shape of the basal callus, the distribution of palp pilosity, and the coloration of the middle tibiae.

**Figure 1:**
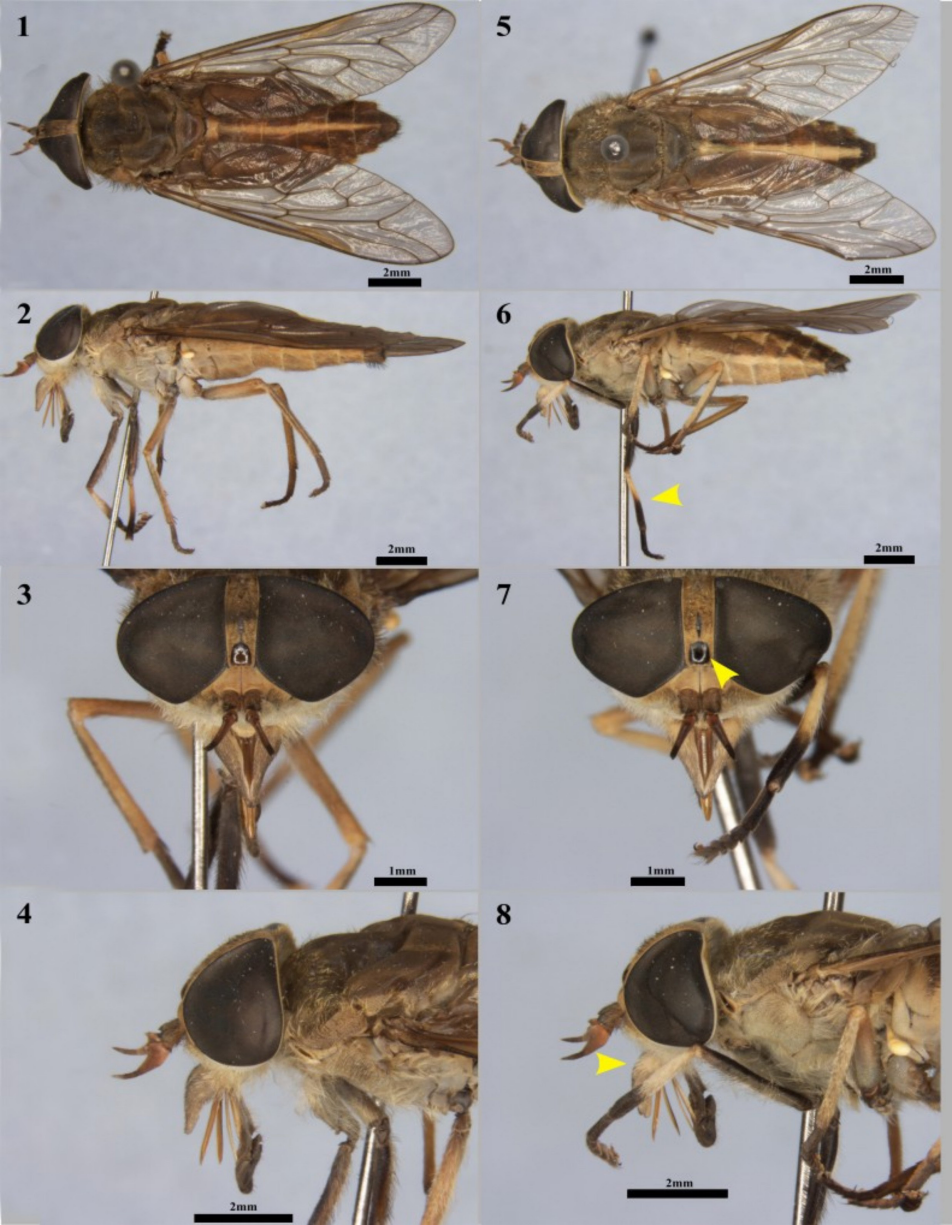
Morphological aspects of *Tabanus occidentalis* (**1-4**) and *T. triangulum* (**5-8**) from the coastal plain of Rio Grande do Sul state. Yellow arrows indicate characters that differ between the two species.

Both *T. occidentalis* and *T. triangulum* can be relevant for pathogen transmission. *Tabanus occidentalis* carries *Trypanosoma* species, possibly *Trypanosoma vivax* (Parra-Henao et al., 2008) and bacteria that can cause opportunistic diseases (Luz-Alves et al., 2007), while *T. triangulum* is a potential mechanical vector of *Trypanosoma kaiowa* (Rodrigues et al., 2021). The distribution of both species overlaps in the Atlantic Forest and Pampa. Thus the correct differentiation *T. occidentalis* and *T. triangulum* through low-cost and user-friendly methods is crucial for developing public policies targeting the control of these species with zoonotic potential.

Geometric morphometrics has been used as an alternative technique to characterize and distinguish groups with morphological similarities using anatomical landmarks (Bookstein, 1991). The advantages of this method include: low cost, high precision (Dujardin, 2008). In the past decade, the application of this technique has expanded to horse flies from various regions, highlighting differences between species that are sometimes indistinguishable by morphological characteristics and barcoding alone (Torres & Miranda-Esquivel, 2016; Changbunjong et al., 2021; Chaiphongpachara et al., 2022; Mullens et al., 2022).

Considering the polymorphism and morphological similarity between *T. occidentalis* and *T. triangulum*, this study aims to differentiate both species, from the coastal plain of the state of Rio Grande do Sul, using geometric morphometrics.

## Materials and Methods

### Study area

**Figure 2:**
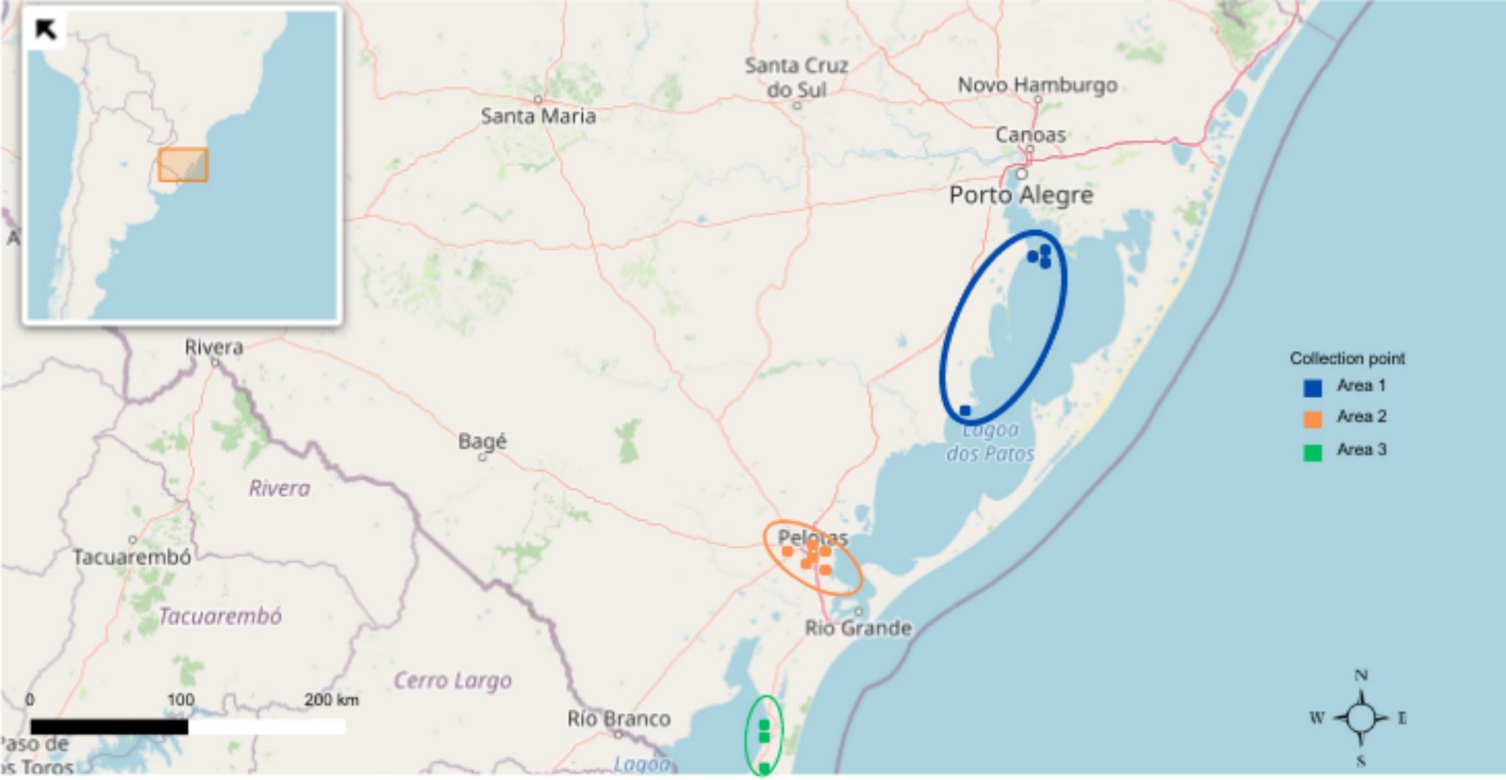
Collection sites of *Tabanus triangulum* and *T. occidentalis* in the coastal plain of Rio Grande do Sul state.

Horse flies were collected using Malaise traps (Townes, 1972) from October 27, 2011, to February 12, 2012, in the coastal plain of Rio Grande do Sul state (CPRS) (see Kirst et al., 2015; Zafalon-Silva et al., 2018). Three collection areas were selected: Area 1: Arroio Pelotas, Arroio Corrientes, and Arroio Grande, characterized by restinga vegetation, wetlands, lowland grassy areas, and sandy regions, distinct from Arroio Pelotas and the Arroio Corrientes have similar vegetation, both conserving remnants of the Atlantic Forest, where pioneer vegetation persists. Collection Area 2: the Lami Biological Reserve, Vila Pacheca on the Rio Camaquã, and the Private Natural Heritage Reserve Barba Negra (RPPN). The Lami area features riparian forests and predominant vegetation composed of shrubby wetlands, herbaceous wetlands, sandy fields, wet grasslands, and forests. Vila Pacheca, on the other hand, hosts primary riparian forest vegetation. A significant part of the RPPN consists of forest vegetation and sandy plains. Collection Area 3: the TAIM Ecological Station (Figure 2, Supplementary Material). This location includes wetlands with palustrine vegetation, coastal fields with savannah-grassy-woody formations, and dune vegetation forming a long stretch of “restinga” (Zafalon-Silva et al., 2018).

### Data collection

The collected horse flies were identified using the methodology described by Silva (2016) and compared to specimens housed in the Entomological Collection of the Federal University of Tocantins (CEUFT). For the identification of the two species, the following characteristics were used (Silva, 2016): the shape of the basal callus, distribution of palp pilosity, and the coloration of the middle tibiae. The coloration of the middle tibiae is darker in *T. triangulum* (Figure 112, 1.6). Additionally, in *T. triangulum*, the callus has a more rectangular shape compared to that of *T. occidentalis* (Figure 113, 1.7). Lastly, *T. occidentalis* exhibits more pilosity at the base of the palps compared to *T. triangulum* (Figure 114, 1.8)

This study used females, which were more frequently captured in Malaise traps (Townes, 1972). The right-wing of collected specimens was extracted and subsequently fixed onto a histological slide and immersed in Enthellan. Following fixation, the wings were photographed using stereoscopes with attached cameras in dorsal view.

**Figure 3:**
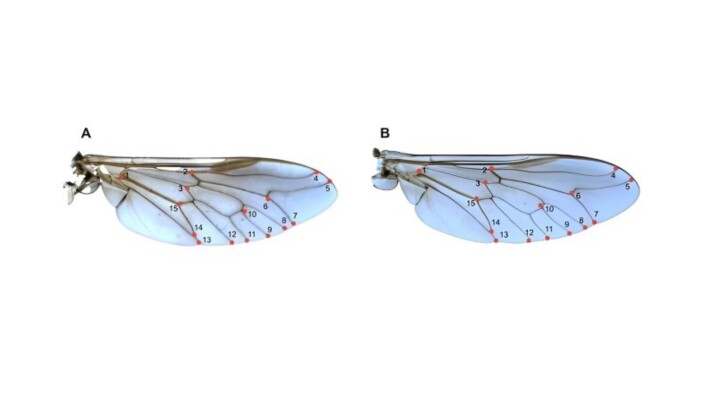
Anatomical landmarks, digitized by TPSDig software, of the right wing of *Tabanus occidentalis* (A) and *T. triangulum* (B) from the coastal plain of Rio Grande do Sul state.

To describe the shape and size of the wings of *T. triangulum* and *T. occidentalis*, 15 type I two-dimensional landmarks were used. For the definitions of the anatomical landmarks, the methodology employed by Torres & Miranda-Esquivel (2016) was followed (Figure 3, Table 1).

**Table 1:** Wing landmarks used in morphometric identification of *Tabanus triangulum* and *T. occidentalis* from the coastal plain of Rio Grande do Sul state.

Landmarks	Position of Points
1	Intersection between veins Cu, M and AR
2	Intersection between veins PR and PR2
3	Intersection between ramifications of vein M
4	Intersection between veins C and PR1
5	Intersection between veins C and PR2A
6	Extremities of vein PR2A and PR2B
7	Extremity of vein PR2B
8	Extremity of vein AM1
9	Extremity of vein AM2
10	Intersection between ramifications of vein M and vein AM2
11	Extremity of vein PM
12	Extremities of vein ACu
13	Extremities of vein A2Cu + AA
14	Intersection between vein AA and A2Cu
15	Intersection between vein PCu and A2Cu

Note: C (costal), Sc (subcostal), AR (anterior radial), PR (posterior radial), PR1 (posterior radial 1), PR1A (posterior radial 1A), PR1B (posterior radial 1B), PR2 (posterior radial 2), PR2A (posterior radial 2A), PR2B (posterior radial 2B), AM (anterior medial), AM1 (anterior medial 1), AM2 (anterior medial 2), PM (posterior, medial), PM1 (posterior medial 1), PM2 (posterior medial 2), A1Cu (anterior cubital 1), A2Cu (anterior cubital 2), PCu (posterior ulnar cubital), AA (anterior anal), PA (posterior anal).

To obtain the coordinates of the anatomical landmarks, the TpsDig software was utilized. To attain a sufficient sampling size to achieve reliable results, 30 individuals from each species were used, as described by Cardini et al. (2015).

### Morphometric geometry

From the obtained anatomical landmarks, a Generalized Procrustes Analysis (GPA) was conducted to superimpose the reference points of all individuals onto a standard coordinate system, removing the effects of translation, rotation, and scaling using least squares (Gower, 1975; Adams et al., 2004).

Subsequently, the general patterns of wing shape variation were obtained, and an alignment of both species was performed to apply the T-test to compare the centroid sizes of the species. Next, a Principal Component Analysis (PCA) was conducted to reduce dimensionality among variables for comparing wing shape patterns and the covariation of *T. occidentalis* and *T. triangulum* (Zelditch et al., 2012).

Multivariate analysis of variance (MANOVA) and canonical variate analysis (CVA) were used to compare the patterns of variation and covariation of the wing shape. In MANOVA, two necessary premises are followed: considering the Euclidean measurement space and ensuring that the number of shape variables is greater than the number of obtained variable coordinates (2K – 4), where ‘K’ is the number of reference points (Dryden & Mardia, 1993; Zelditch et al., 2012). The primary objective of MANOVA is to ascertain shape differences between classes of independent variables. In this instance, the difference between the means of variables obtained through the principal components it assessed. With CVA, differences between *T. triangulum* and *T. occidentalis* from the CPRS were examined based on newly generated axes called canonical variables (CV) to maximize group differences (Cooke & Terhune, 2015). For this, a 1000-times bootstrap was utilized. All analyses were conducted using R Core Team 4.3.3 (R Core Team, 2023) software with the '*Geomorph*' (Adams et al., 2024), '*Vegan*' (Oksanen et al., 2022), and '*Tidyverse*' (Wickham et al., 2019) packages.

## Results

**Figure 4:**
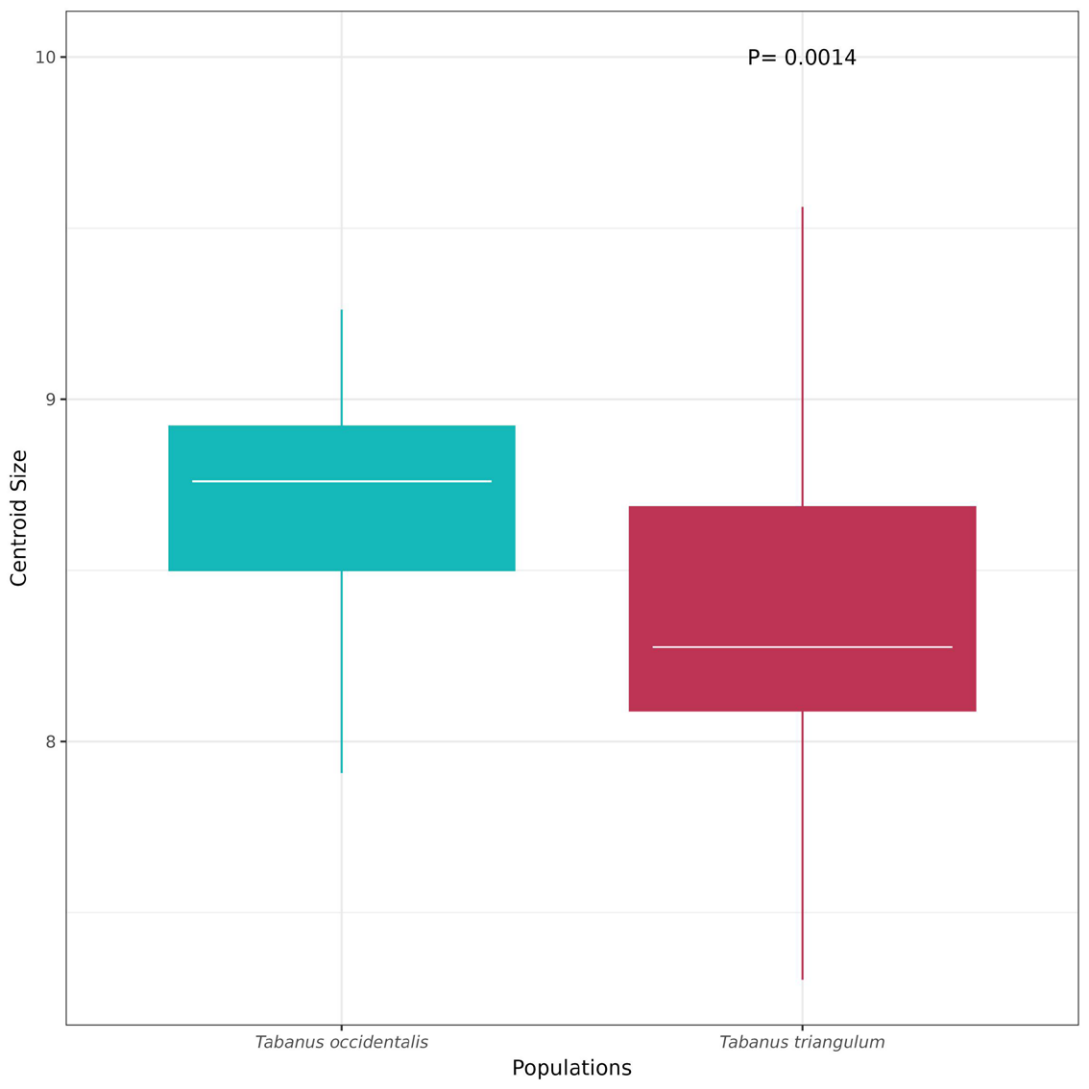
Boxplot of centroid size of the wings of *Tabanus occidentalis* and *T. triangulum* from the coastal plain of the state of Rio Grande do Sul.

The average centroid size calculated for *T. occidentalis* was 8.700 mm ± 0.305, and for *T. triangulum*, it was 8.330 mm ± 0.515 (Figure 4). Therefore, based on the centroid sizes, there was a significant difference between the two species (P= 0.001).

The morphospace is formed by PC1 (20% of the total variance) and PC2 (15% of the total variance) with a cumulative proportion of 35%. The MANOVA indicated that *T. occidentalis* and *T. triangulum* differ in wing shape (Table 2). Although the two species can also be distinguished based on centroid size (Table 2 and previous analysis), the interaction between centroid size and wing shape was not statistically significant. Thus, both species exhibited the same allometric trajectories with shape variation without size differences (P= 0.646).

**Table 2:** Multivariate analysis of variance for 15 wing landmarks used in morphometric identification of *Tabanus triangulum* and *T. occidentalis* from the coastal plain of Rio Grande do Sul state.

	Df	Wilks	F	Residuals	P
Species	1	0.246	8.597		<0.001
Centroid Size	1	0.576	2.065	56	0.033
Species x Centroid Size	1	0.773	0.825		0.646

Df: degrees of freedom; Wilks: test applied. F: approximate F value, P: P value.

**Figure 5:**
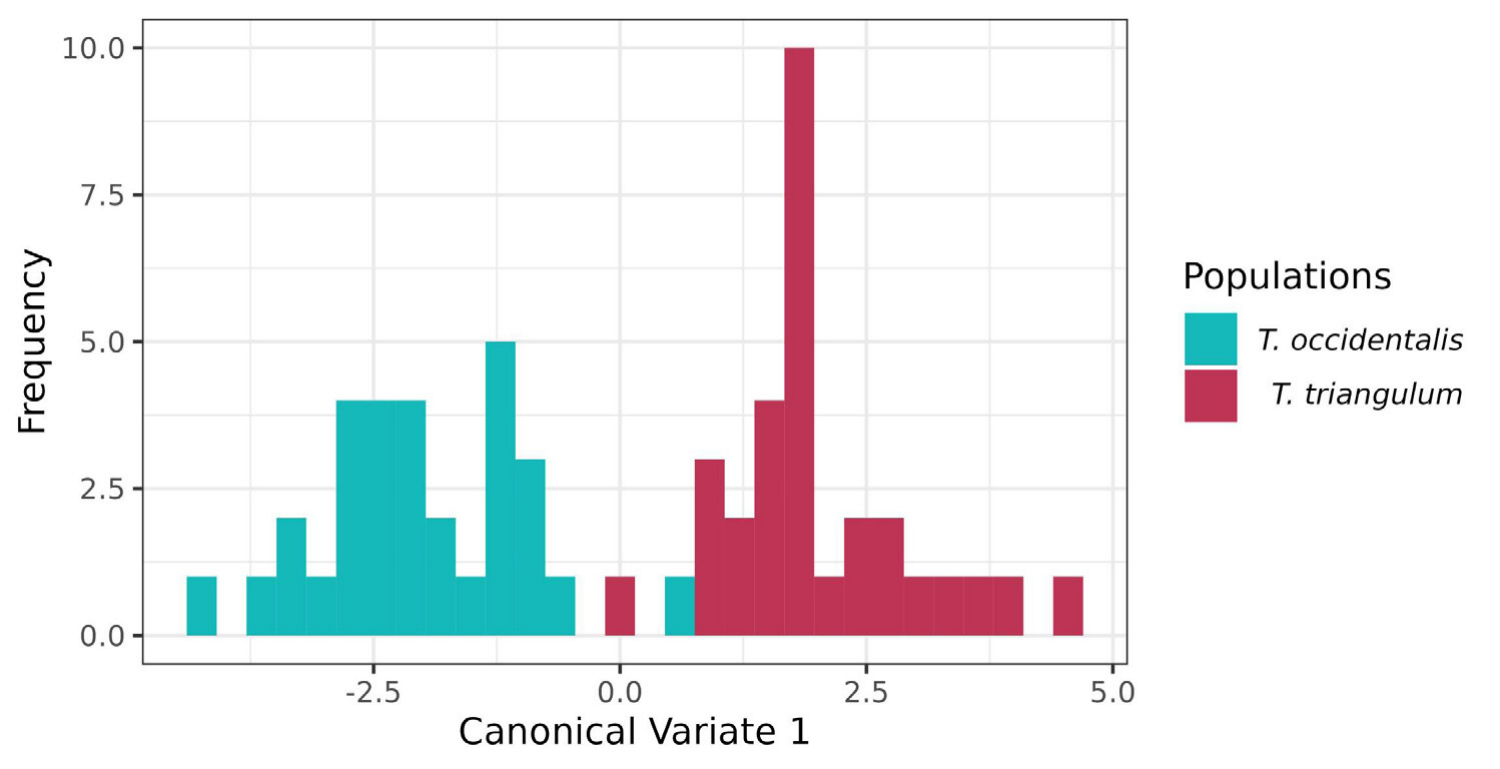
Canonical Variates Analysis (CVA) showing the difference between the wings of *T. triangulum* and *T. occidentalis*.

The canonical variance analysis (CVA) of wing shape produced distinct clusters for the species (Figure 5). The CVA results for Tabanidae demonstrated substantial segregation between *T. triangulum,* with an accuracy of 83,33% and, *T. occidentalis* with an accuracy of 76,67%) with an overall classification accuracy of 80% and a Mahalanobis distance of 4.02 (P = 0.001).

**Figure 6:**
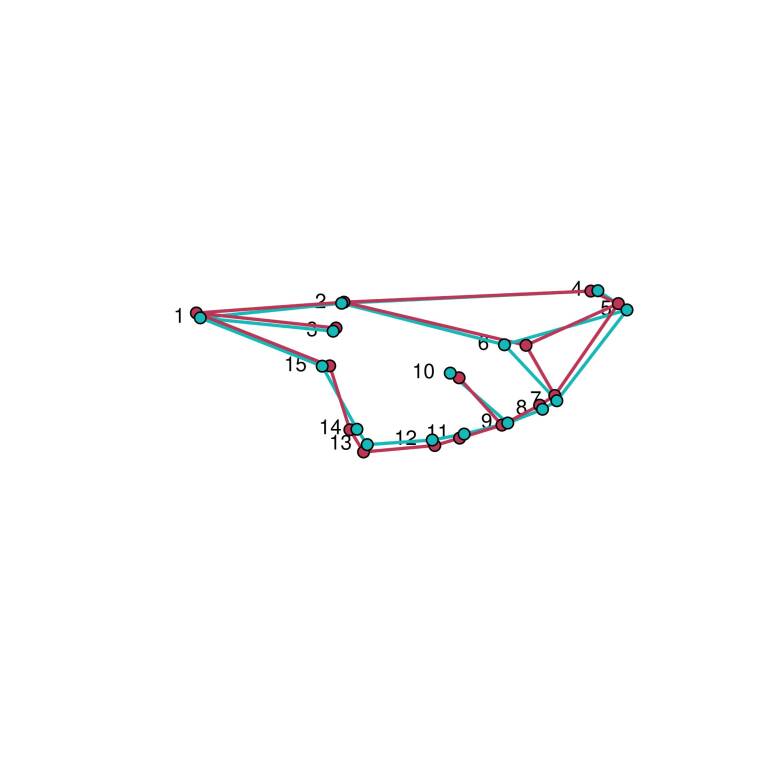
Mean shape of the wings based on the 15 highlighted points on the wings of *T. triangulum* (magenta) and *T. occidentalis* (cyan).

The CVA results showed that *T. occidentalis* has a more elongated wing, as evidenced by the shifts in landmarks 5, 6, and 7. At the same time, landmark 15 moved posteriorly towards the base of the wing, making it more tapered. Additionally, landmarks 7 and 9 movements brought the veins closer at the tip of veins MA1 and MA2. The opposite trend was observed for *T. triangulum*: this species has a sturdier wing with a slightly angled base. The distal portion of the wing is more angled, as suggested by a shift in landmark five posteriorly, and landmark six towards the apex of the wing, which reduces cell size at the intersection with veins C and RP2A and the intersection with veins RP2A and RP2B (Figure 6). The changes observed in anatomical landmarks 13, 14, and 15 make the wing bases of *T. triangulum* and *T. occidentalis* very distinct, making the wing base sturdier in *T. triangulum* than in *T. occidentalis*. These changes in the anatomical landmarks of the wing base alter the membranous areas of the anal lobe, anal and posterior regions.

## Discussion

In this study, it was demonstrated that the GM approach based on landmarks efficiently distinguishes between *T. occidentalis* and *T. triangulum* in the Pampa. Additionally, adaptations to the environment and the relationship between the wing shape of each species with size and function in flight behavior can be explored.

Various authors have applied GM to discriminate horse fly species worldwide (Chaiphongpachara et al., 2022; Mullens et al., 2022). Their findings revealed that methods based on landmarks and contours can distinguish between Tabanidae species. In the present study, 15 anatomical landmark positions were selected according to the study of Torres & Miranda-Esquivel (2016). These positions distinguished between *T. occidentalis* and *T. triangulum*. They can also be used to investigate the phenotypic variation of the group (Torres & Miranda-Esquivel, 2016; Mullens et al., 2022; Changbunjong et al., 2021), further allowing inferences about the functional morphology of the wings of these two species.

The wing of *T. triangulum* appeared smaller and sturdier than that of *T. occidentalis*, which is slightly more tapered with more significant modifications in the relationship of the radial veins 4 and 5 at the posterior tip and the veins in the anal region. These changes might be associated with shifts in the aerodynamic properties of the wings, as Belyaev & Farisenkov (2019) argued for insects. Changes in the radial ribs, which terminate on the posterior margin of the wing, can cause an increase in wing length from the base to the tip, allowing the fly to achieve faster speeds (Nabawy & Crowther, 2015, 2016). This suggests that *T. occidentalis* can fly faster than *T. triangulum,* since there is less drag movement in the case of more tapered wings (Walker, 2002). It is advantageous for larger insects to have long and narrow wings due to the peculiarities of vortex formation attached to the leading edge (Harbig et al., 2013). However, long and narrow wings are not always correlated with body mass (Belyaev et al., 2012).

The sturdier wings of *T. triangulum* tend to favour more stable flight but with fewer possibilities for changes in direction (Bhat et al., 2019; Krishna et al., 2020). More robust and stable wings benefit take-off, landing, and flight, especially at lower altitudes where greater air densities (fluid) exert greater forces on the wing (Dudley, 2002). In the coastal plain of the low altitudes of Rio Grande do Sul there are strong south-north oceanic winds, and high humidity. In this region, sturdier wings will be less affected by wind gusts due to their larger surface, providing more stability (Dudley, 2002).

Wings that are large with respect to body dimensions, like those of *T. triangulum* and *T. occidentali*s, favor high speeds and high lift capacity in relation to drag forces. They also help the feeding behaviour of female horse flies on vertebrates. Female horse flies perform telmophagy (Mullens, 2019); that is, they induce blood leakage for feeding, causing discomfort to the host and swift reactions. Consequently, the flies must sustain their flight for long periods, and sustaining flight demands robust, large wings that achieve high speeds and acceleration rates. This allows the fly to locate and pursue its hosts or to quickly switch to a nearby host (Barros & Foil, 2007).

Moreover, the behavior of horse flies requires great flight control capacity, especially during high sun exposure, since they are diurnal insects (Kriska et al., 2009) that can dehydrate during peak temperature hours (Burnett & Hays, 1974; Schutz & Gaugler, 1992). Therefore, open, sunny areas with consistent winds throughout the year, like the livestock fields in the Pampa biome and coastal plains, may favour species that are adapted to cope with these conditions, as is the case of *T. triangulum*.

The differences in the shape and size of the wing of *T. triangulum* and *T. occidentalis* in southern Brazil might not be explained solely by the differences in one of their locomotive structures. Wing geometric variations alone cannot explain the high abundance of *T. triangulum* in the region (Silva, 2016). Other factors might be involved in the great numbers of *T. triangulum* on the coastal plains and in the Pampa of Rio Grande do Sul, such as thermal limits for the development of immatures (Mullens, 2019), availability food resources for males (Kniepert, 1980), historical factors (Cranston, 2005), or even the latitudinal gradient (Chown & Gaston, 2010).

Allen's rule dictates that wing size correlates with the size of a species (Frӧhlich et al., 2023). One of the conclusions of this study is that this rule applies to *T. triangulum* and *T. occidentali*s. Considering the latitudinal pattern, species that occur and are dominant in colder climates, like *T. triangulum*, tend to be more robust. This is confirmed by looking at the distribution of both species, which can occur concomitantly in the eastern part of the south (Bassi et al., 2000; Silva, 2016) and southeast (Guimaraes et al., 2016) of Brazil. In these locations, *T. triangulum* displays high relative abundance, representing more than 70% of all specimens collected from many localities of the coastal plains of Rio Grande do Sul (Krüger & Krolow, 2015; Silva, 2016). *T. occidentalis*, is limited to the south, in the same localities where *T. triangulum* occurs, but with a much lower relative abundance (Silva, 2016), becoming more frequent in warmer localities to the north of the southern region (Bassi et al., 2000) and on the coast of the southeastern region, where both species co-occur (Guimaraes et al., 2016). This presents an intriguing process for future studies on the functional morphology and geographic variation of horse fly wings in the Neotropics.

The GM approach based on landmarks showed 80% accuracy in distinguishing *T. triangulum* and *T. occidentalis*. Thus, the landmark coordinate data of this study can be used to perform the morphometric identification of these species. Furthermore, it is suggested that a landmark-based GM approach can complement traditional morphological identification, especially when specimens are difficult to identify. The GM approach is a reliable tool for identifying cryptic species of Tabanidae and can lead to more effective control measures for horse flies in livestock farms.

## Supplementary Material

Supplementary material accompanies this paper.

Legend S1 Location of collection points

This material is available as part of the online article from https://doi.org/10.1590/S1984-29612024028
