# Supplementary material for: Discrimination of cryptic species: Tabanus triangulum and Tabanus occidentalis (Diptera: Tabanidae) differ in size and shape
Source: Rev Bras Parasitol Vet. 2024 Jun 17;33(2):e020123. doi: 10.1590/S1984-29612024028 (PMC11253824; doi:10.1590/S1984-29612024028)
Supplement: Legend S1 [file rbpv-33-2-e020123-Suppl.pdf]

Local	Decimal	Decimal
**Region 1**	-52.25422	-31.72175
	-52.21764	-31.67118
	-52.26732	-31.64843
	-52.14568	-31.55675
	-52.13951	-31.56435
	-52.12173	-31.43295
	-52.00834	-31.49678
**Region 2**	-52.25422	-31.72175
	-52.21764	-31.67118
	-52.26732	-31.64843
	-52.14568	-31.55675
	-52.13951	-31.56435
	-52.12173	-31.43295
	-52.00834	-31.49678
**Region 3**	-52.50060	-32.55568
	-52.50975	-32.56059
	-52.52538	-32.53347
	-52.53713	-32.53845
	-52.48655	-32.63511
	-52.50762	-32.56238
	-52.55767	-32.56818
